# The Hepatitis *Delta* Virus accumulation requires paraspeckle components and affects *NEAT1* level and PSP1 localization

**DOI:** 10.1038/s41598-018-24500-1

**Published:** 2018-04-16

**Authors:** Yasnee Beeharry, Gabrielle Goodrum, Christian J. Imperiale, Martin Pelchat

**Affiliations:** Department of Biochemistry, Microbiology and Immunology, Faculty of Medicine, University of Ottawa, Ottawa, Ontario, K1H 8M5 Canada

## Abstract

The Hepatitis *Delta* Virus (HDV) relies mainly on host proteins for its replication. We previously identified that PSF and p54nrb associate with the HDV RNA genome during viral replication. Together with PSP1, these proteins are part of paraspeckles, which are subnuclear bodies nucleated by the long non-coding RNA *NEAT1*. In this work, we established the requirement for PSF, p54nrb and PSP1 in HDV replication using RNAi-mediated knockdown in HEK-293 cells replicating the HDV RNA genome. We determined that HDV replication induces the delocalization of PSP1 to cytoplasmic foci containing PABP and increases *NEAT1* level causing an enlargement of *NEAT1* foci. Overall, our data support a role for the main paraspeckles proteins in HDV life cycle and indicate that HDV replication causes a cellular stress and induces both a delocalization of the PSP1 to the cytoplasm and a disruption of paraspeckles.

## Introduction

The Hepatitis *Delta* Virus (HDV) is composed of a small negative single-stranded circular RNA genome of approximately 1700 nucleotide that self-folds and adopts a rod-like structure^1,2^. Although it requires the hepatitis B virus (HBV) envelope proteins for encapsidation and dissemination^3^, HDV relies mainly on its host proteins for replication and replicates without HBV. Replication of the HDV RNA genome takes place in the nucleus, and occurs by a symmetrical, rolling circle mechanism^4^. Replication of the infectious circular monomer produces linear, multimeric strands, which are subsequently cleaved by endogenous ribozymes and ligated, yielding antigenomic circular monomers. Using antigenomic circular RNA monomers, the same three steps are repeated to generate genomic circular RNA monomers. During this process, a viral mRNA encoding a single open reading frame is also produced. Due to editing of antigenomic HDV RNA during viral replication^5–7^, the HDV mRNA can produce two proteins (i.e. HDAg-S and HDAg-L). The small antigen (HDAg-S; 195 amino acids) is required for HDV accumulation^8,9^, and the large antigen (HDAg-L; 214 amino acids) is involved in the viral encapsidation by the HBV envelope proteins^10,11^.

The HDV RNA genome uses host RNAP II for both its replication and transcription. HDAg mRNA has a 5′-cap and a 3′-poly(A) tail, features found on transcripts generated by RNAP II^12,13^. Low concentrations of α-amanitin, known to inhibit RNAP II, block the accumulation of both HDAg mRNA and genomic HDV RNA in cultured cells as well as in nuclear extracts^14,15^. RNAP II association with both polarities of the RNA genome further confirmed a role for this polymerase in the life cycle of HDV^16–18^. Specifically, co-immunoprecipitations, binding assays, mutagenesis and *in vitro* transcription experiments indicated that RNAP II interacts with sites located within the tips of the terminal stem-loop domains of both polarities of HDV RNA^16,17,19–22^. To obtain insight into the recognition of HDV RNA promoters by RNAP II, we previously investigated the transcription complex forming on a fragment acting as an RNA promoter for RNAP II and containing the transcription initiation site for HDAg mRNA^17,19,22^. In addition to RNAP II subunits typically used for DNA promoter recognition (i.e. RNAP II, TFIIA, TFIIB, TFIID, TFIIE, TFIIF, TFIIH, and TFIIS)^17^, we identified that several proteins having roles in RNA-processing pathways associate with HDV RNA^23,24^.

Among these proteins, the polypyrimidine tract-binding protein-associated splicing factor (PSF) was identified as an HDV RNA-binding protein^23,24^. The interaction between PSF and both polarities of HDV RNA was demonstrated by co-immunoprecipitation experiments using a monoclonal antibody specific for PSF, both *in vitro* using HeLa nuclear extract and within HeLa cells containing both polarities of the HDV genome^24^. Furthermore, binding of purified recombinant His-tagged PSF to various HDV-derived RNAs confirmed the specificity of the interaction, and indicated that the protein binds directly to fragments corresponding to the terminal stem-loop domains of both polarities of HDV RNA^24^. Because these fragments also include sites where RNAP II interacts with and initiates transcription from HDV RNA^12,15–19^, it was suggested that PSF might have a role in RNAP II-mediated HDV replication^24^.

PSF is a multifunctional protein that belongs to the Drosophilia Behaviour Human Splicing family of proteins (DBHS)^25,26^. PSF can act independently or form a heterotetramer with the 54-kDa nuclear RNA-binding protein (p54nrb), a protein that bears significant homology to the C-terminal domain of PSF^26^, and that we also identified as an host protein associating with HDV RNA during replication^23^. These two proteins facilitate a number of nuclear activities including splicing, polyadenylation, transcriptional regulation, retention of defective RNAs, nucleic acid unwinding and annealing, nuclear shuttling, and DNA recombination^26^. Similarly to PSF and p54nrb, the Paraspeckle Protein 1 (PSP1) is also a DBHS protein but its link with HDV replication has not been previously investigated^27^. PSF, p54nrb and PSP1 are found ubiquitously in the nucleoplasm and in the nucleolar caps, and are present in higher concentrations in paraspeckles^26,28–30^.

Paraspeckles are small foci situated at the periphery of the nuclear speckles and their number range between 2 to 20 per cell^28,31–35^. They are composed of several proteins nucleated around the long non-coding RNA Nuclear Enriched Associated Transcript 1 (*NEAT1*). *NEAT1* is proposed to fold into a rod-like structure and serves as a scaffold for recruiting DBHS proteins^36^. Paraspeckles were suggested to act as storage for A-to-I edited RNAs and for several proteins associated with transcriptional repression^37,38^. Paraspeckles may also be involved in the cellular response to stress, as it was reported that paraspeckles form upon stress shock and interferon stimulation, and that cellular stresses induce NEAT1 transcription^36,39^. Paraspeckles were also reported to have a role in viral infection, such as with the Human Immunodeficiency Virus, Epstein Barr Virus, Influenza A Virus and Herpes Simplex Virus^40–45^. However, it remains unexplored whether there is a link between HDV replication and paraspeckles.

Because the HDV RNA genome interact with PSF and p54nrb, two of the main proteins constituting paraspeckles, it is possible that HDV uses paraspeckle components for its replication, and consequently might disrupt paraspeckles formation. In this study, we used RNAi-mediated knockdown of PSF, p54nrb and PSP1 in cells replicating HDV RNA to determine the requirement for these proteins in HDV replication. Using immuno-staining, fluorescence *in situ* hybridization (FISH) and cellular fractionation, we investigated the effect of HDV replication on the localization of PSF, p54nrb, PSP1 and *NEAT1*. Furthermore, because stress shock caused by viral infection could affect *NEAT1* accumulation, we also quantified *NEAT1* levels in cell replicating the HDV RNA genome.

## Results

### PSP1 associates with the HDV RNA genome in HEK-293 cells

Previously, we demonstrated that PSF and p54nrb associate with the HDV RNA in cells replicating the HDV genome^23,24^. These two proteins and PSP1 are three of the main components of nuclear paraspeckles^26,28,29,46^. To determine whether PSP1 also interacts with the HDV RNA genome, we performed RNA-protein immunoprecipitation (RIP) assays for this protein in cells replicating HDV. For this, we used a cellular system where HEK-293 cells were stably transfected with a plasmid encoding HDAg-S under the control of a promoter activated by tetracycline and with an HDV RNA genome unable to produce HDAg (i.e. 293-HDV)^47^. Twenty-four hours after induction of HDAg-S expression with 1 μg/ml of tetracycline, and thus induction of HDV replication, the cells were treated with formaldehyde to cross-link the RNA-protein complexes. Following disruption of the cells, the ribonucleoprotein complexes were immunoprecipitated with an α-PSP1 antibody, or with either α-PSF or α-p54nrb antibodies as positive controls. As a negative control, α-β-actin was used as an unrelated antibody.

**Figure 1:**
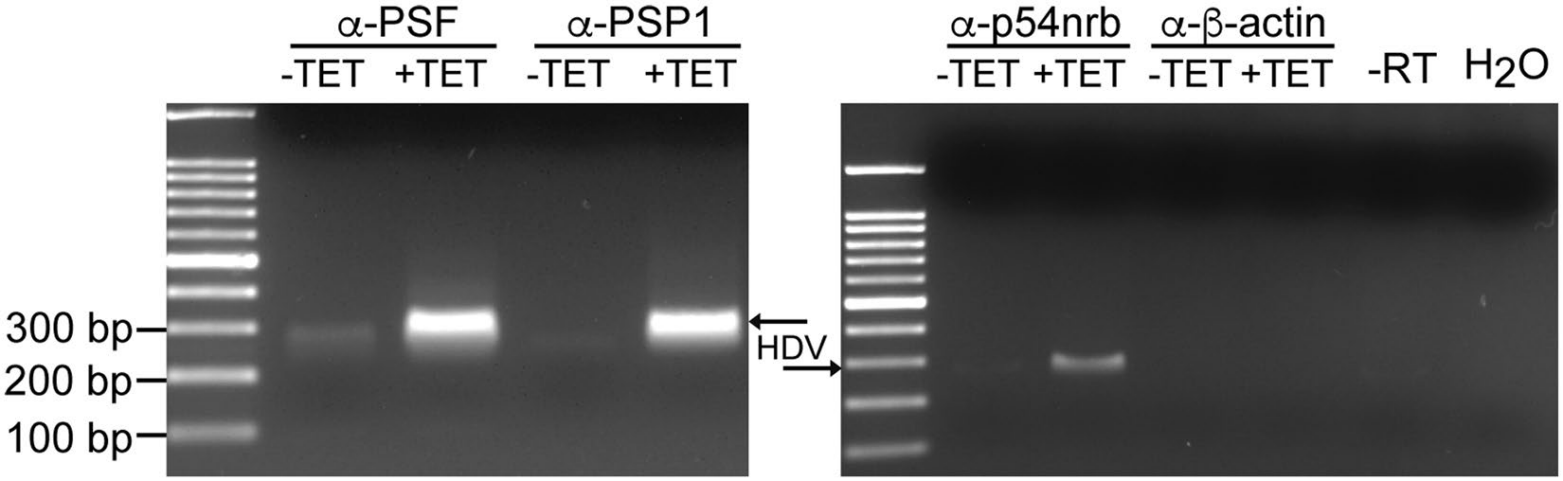
Association of HDV RNA with PSF, p54nrb and PSP1 in HEK-293 cells. HDV replication in 293-HDV cells was induced (+) or not (−) by tetracycline, then the cells were treated with formaldehyde to cross-link the RNA-protein complexes and lyzed. The lysates were used for RIP using α-PSF, α-p54nrb and α-PSP1 antibodies. The α-β-actin antibody was used for RIP as a negative control. Following co-immunoprecipitation, the cross-links were reversed by heating the samples and RT-PCR was carried out to amplify a 300 bp fragment of the HDV RNA genome located on the terminal domain containing the ribozyme sequences. The resulting PCR product was resolved on an agarose gel. The 100 bp DNA Ladder (NEB) was used as marker.

Following the co-immunoprecipitations, the cross-links were reversed by heating the samples and RT-PCR was carried out to amplify a fragment of the HDV RNA genome corresponding to the terminal domain containing the ribozyme sequences. When the co-immunoprecipitations were performed with either α-PSP1, α-PSF or α-p54nrb antibodies, we observed the presence of a band of the expected length for the HDV amplicon in the cells treated with tetracycline (Fig. 1). The band observed was not due to a non-specific binding of the RNA to the protein G agarose beads or to antibody constant regions, because this amplicon was not observed in the negative controls. Taken together, our results indicate that PSP1 associates, directly or indirectly through protein:protein or protein:RNA:protein interactions, with the HDV RNA genome in this cellular system, and corroborate our previous observations of the association of both PSF and p54nrb with HDV RNA^23,24^.

### Knockdown of PSF, p54nrb and PSP1 leads to an inhibition of HDV replication

**Figure 2:**
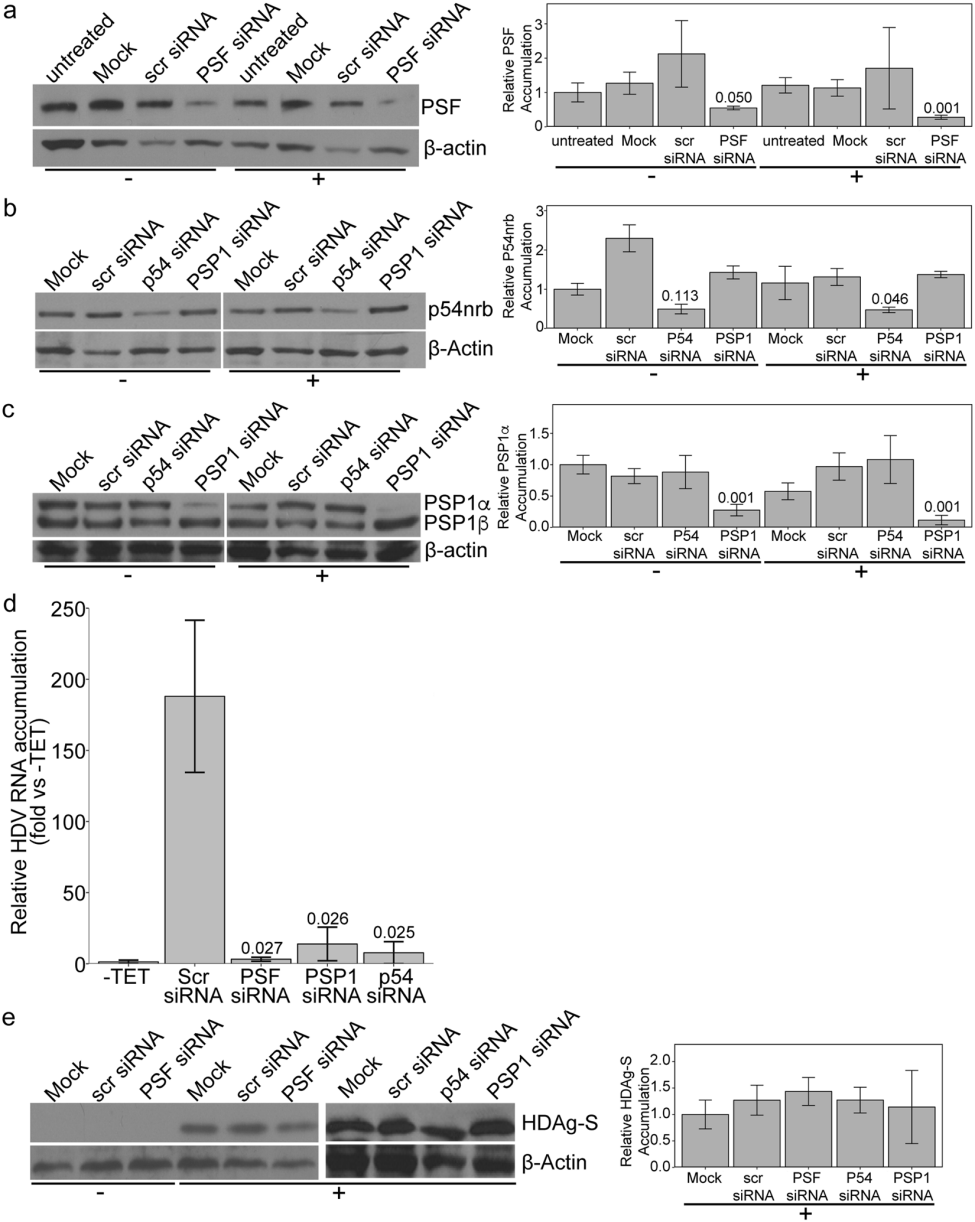
Knockdowns of PSF, p54nrb and PSP1 reduce HDV RNA genome accumulation. 293-HDV cells were transfected with pools of siRNAs against either PSF mRNA, PSP1 mRNA, p54nrb mRNA, or a pool of five scrambled siRNAs (Scr). Transfection with water (Mock) or untransfected cells (untreated) were used as controls. Twenty-four hours post-transfection, the expression of HDAg-S, and thus HDV replication, was induced (+) or not (−) with tetracycline. Twenty-four hours post-induction, total RNA and protein were extracted. (**a**–**c**) Western blotting and quantification of the bands showing the relative amount of PSF (**a**), PSP1 (**b**) and p54nrb (**c**) in each treatment compared to β-actin. (**d**) Quantification by RT-qPCR of the HDV RNA genome levels normalized to the amount of β-2-microglobulin mRNA and to the untreated cells. Values represent the mean and standard deviation of three biological replicates. Unpaired two-tailed t-tests between the three treatments and the cells induced with tetracycline and transfected with scrambled siRNAs were performed (p-values are indicated above the bars). (**e**) Western Blotting and quantification of the bands showing the relative amount of HDAg-S compared to β-actin following knockdowns of PSF, PSP1 and p54nrb.

To clarify the involvement of PSF, p54nrb and PSP1 in HDV replication, we investigated whether knockdown of these proteins would affect HDV accumulation following induction of HDAg-S production. 293-HDV cells were transfected with commercial pools of siRNAs targeting mRNAs coding for PSF, p54nrb or PSP1. As negative controls, we also transfected cells with scrambled siRNAs or only water. Twenty-four hours post-transfection with the siRNAs, the expression of HDAg-S, and thus HDV replication, was induced by addition of 1 μg/ml tetracycline. Twenty-four hours post-induction, total cellular lysates were prepared and separated in two samples: one was used for protein-extraction and the other was used for RNA extraction. Under our conditions, PSF, p54nrb and PSP1 were significantly knocked down when compared to the cells transfected with scramble siRNAs (Fig. 2a–c).

The effects of the knockdown of these three proteins on the accumulation of HDV RNA was assessed by quantitative RT-PCR (RT-qPCR) using the level of β-2-microglobulin mRNA to normalize the results. As expected when HDV replication was induced with the addition of tetracycline, we observed about 200-fold increase of HDV RNA compared to the untreated cells (Fig. 2d). Upon PSF, p54nrb or PSP1 knockdown, we observed a decrease of more than 90% of HDV RNAs accumulation compared to the cells only treated with tetracycline (Fig. 2d). In order to rule out the possibility that this effect could result from a reduced HDAg-S levels, which is required for HDV replication, we performed a Western Blot on HDAg-S. There was no substantial difference in HDAg-S levels in cells transfected with the siRNAs targeting mRNAs coding for PSF, p54nrb or PSP1 compared to cells transfected with the pool of scramble siRNAs in cells replicating HDV (Fig. 2e). In summary, the knockdown of PSF, p54nrb and PSP1 leads to a decrease of the HDV genome accumulation, indicating that these proteins are required directly or indirectly for the accumulation of HDV genomes in these cells.

### HDV replication induces the formation of PSP1 foci outside of the nucleus

**Figure 3:**
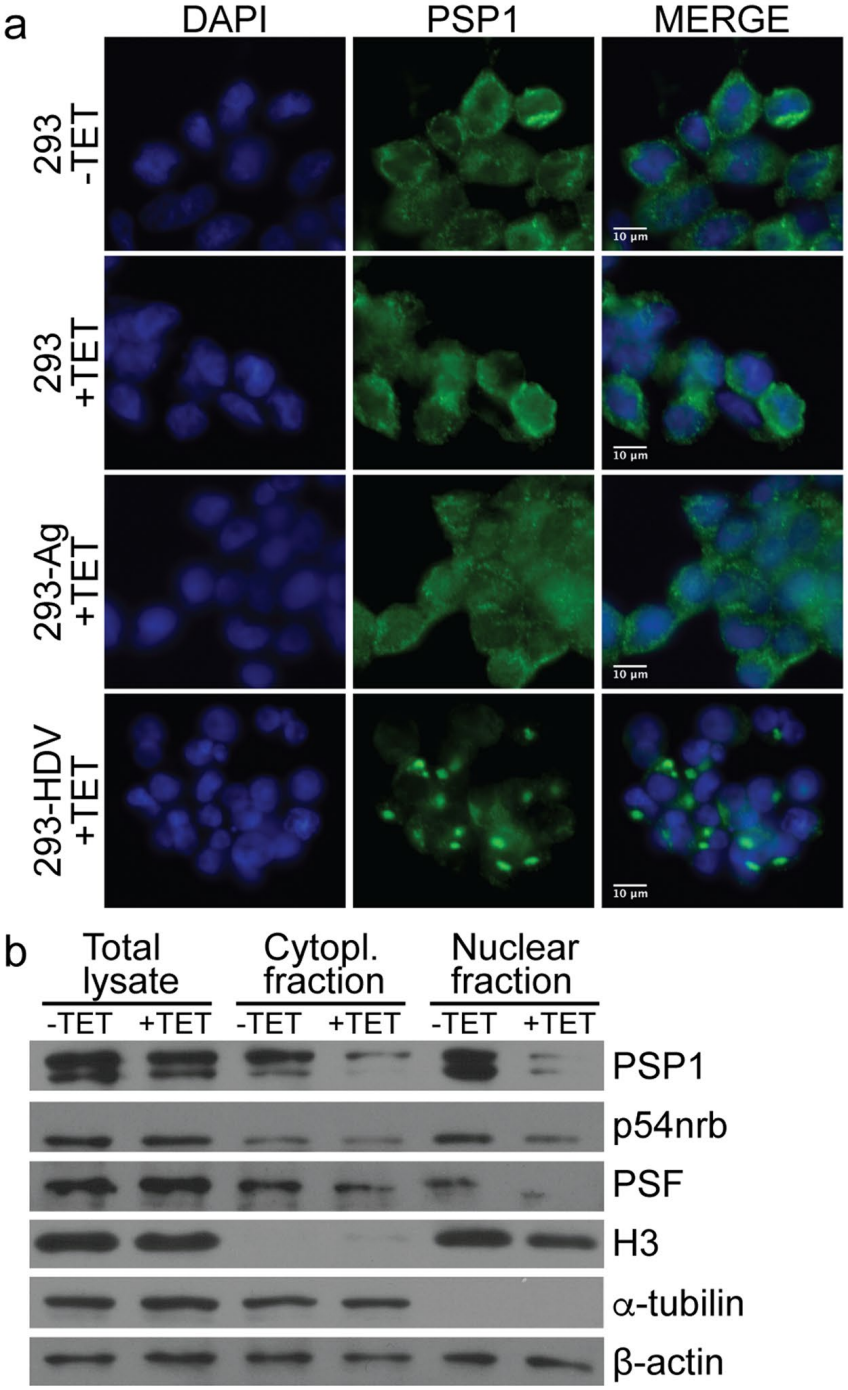
HDV replication induces the formation of PSP1 foci in HEK-293 cells. (**a**) Immunostaining of 293, 293-Ag and 293-HDV cells treated with tetracycline using antibodies against PSP1 (green). DAPI (blue) was used to visualize the nucleus. (**b**) Presence of PSP1, p54nrb and PSF within sub-cellular fractions of the 293-HDV cells treated (+) or not (−) with tetracycline. α-tubulin and α-H3 antibodies were used as cytoplasm and nucleus markers, respectively.

Since the HDV RNA genome interacts with at least three proteins found in paraspeckles (PSF, p54nrb and PSP1), and that these proteins are required for the accumulation of HDV RNA, we hypothesized that paraspeckles might be disrupted upon HDV replication. Since PSP1 has often been used as marker for paraspeckles^28^, we investigated the localization of this protein upon induction of HDV replication. Briefly, HEK-293 cells (293), HEK-293 cells stably transfected with a plasmid encoding the HDAg-S under the control of a promoter inducible by tetracycline (293-Ag), and 293-HDV cells were treated or not with 1 μg/ml tetracycline. Forty-eight hours after this treatment, we performed an immuno-staining using an antibody directed against the C-terminal domain of PSP1. Upon induction of HDV replication, we observed that PSP1 delocalizes outside of the nucleus as large bright foci (Fig. 3a). This pattern was not observed in 293 cells nor in 293-Ag cells treated with tetracycline.

To confirm these results using a complementary approach, we performed sub-cellular fractionations of the cells replicating HDV, followed by a Western Blot for PSP1. The nuclear and cytoplasmic fractions were properly separated and validated using α-tubulin and α-H3 antibodies as cytoplasm and nucleus markers, respectively. In the samples where HDV replication was induced, we observed lower level of PSP1 in both cellular sub-fractions (Fig. 3b). Because H3, α-tubulin and β-actin levels were not significantly reduced by the cellular fractionation, and that PSP1 levels were not greatly affected by the induction of HDV replication (Fig. 3b, see Total lysate), our results suggest the presence of PSP1 into insoluble aggregates. In order to determine if the localization of the other two paraspeckles proteins, PSF and p45nrb, are similarly affected by HDV replication, we repeated the sub-cellular fractionation experiments described above for PSF and p54nrb. In contrast to PSP1, we observed no substantial changes in the localization of p54nrb when HDV replication was induced (Fig. 3b). Interestingly, we observed that induction of HDV replication promotes PSF cytoplasmic localization (Fig. 3b), which was previously shown to be linked to cell cycle arrest^48^.

**Figure 4:**
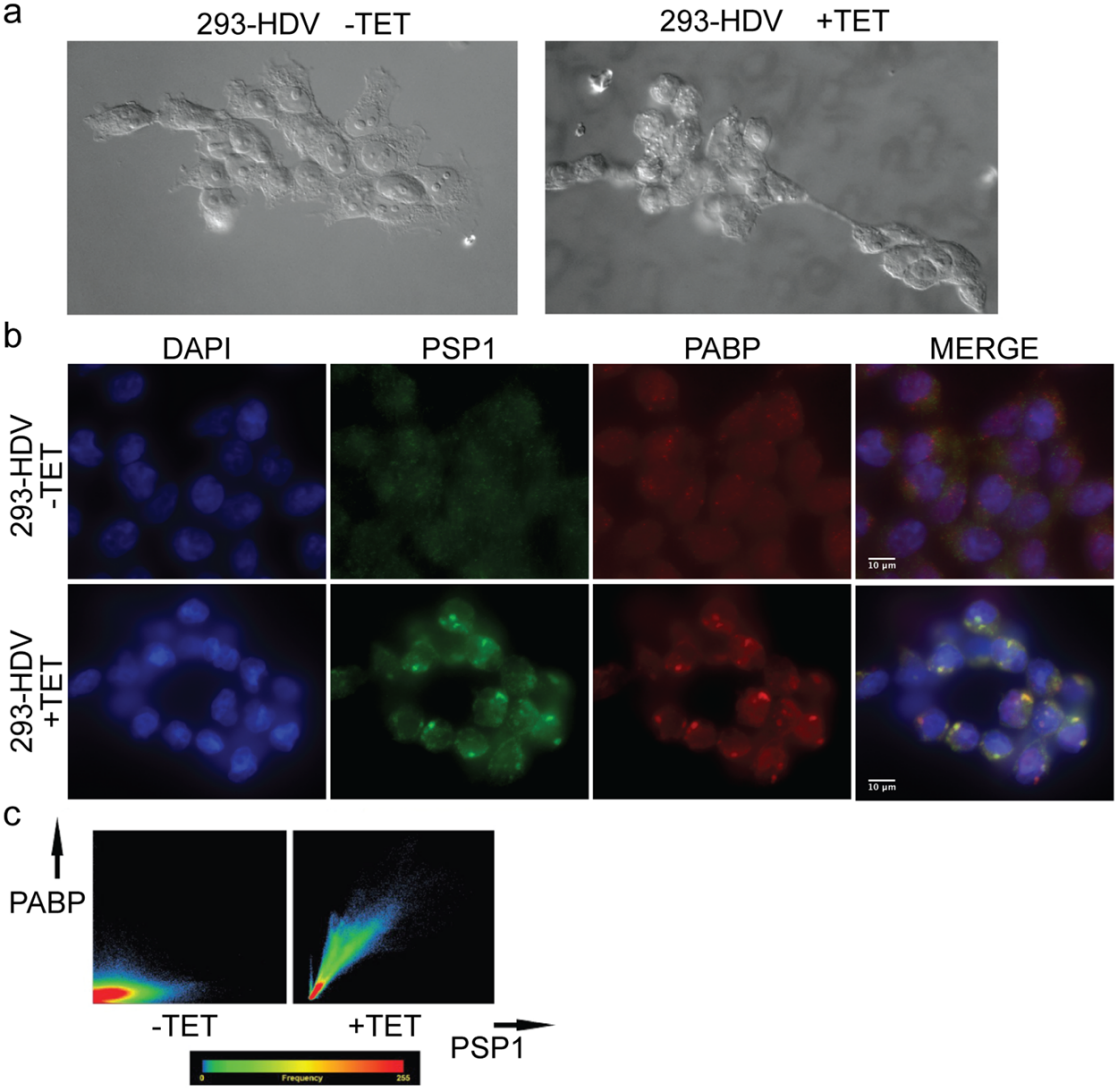
Co-localization of PSP1 with PABP. (**a**) Aspect of the 293-HDV cells treated or not with tetracycline observed under a microscope bright light. (**b**) 293-HDV cells treated or not with tetracycline were immunostained with antibodies against PSP1 (green) and PABP (red). DAPI (blue) was used to visualize the nucleus. (**c**) Quantification by heatmap representations of PABP (y axis) PSP1 (x axis) co-localization.

We and others observed morphological changes in the 293-HDV cells when HDV replication was induced: the cells were round shaped compared to the uninduced cells and detached very easily from the culture dish surface after more than two days post induction (Fig. 4a)^47^. It was also reported that HDV induction causes a significant cell cycle arrest in G1/G0^47^. Given the phenotype, the cellular cycle arrest reported, the PSF cytoplasmic localization, and our results suggesting that PSP1 is present in insoluble aggregates, one hypothesis is that HDV replication could cause a cellular stress and that such a stress might induce the delocalization of PSP1 to the cytoplasmic stress granules. To investigate this hypothesis, we immuno-stained PSP1 and the stress granule marker PABP with or without induction of HDV replication^49^. Upon induction, we observed a strong co-localization of the immunofluorescence signal corresponding to both PABP and PSP1 (Pearson coefficient of 0.94 and Manders coefficient of 0.97; Fig. 4b and c). In contrast, we did not observe this effect in non-induced cells. This result suggests that HDV replication cause a delocalization of PSP1 to PABP-containing stress granules in this cellular system.

### HDV replication decreases PSP1 co-localization with *NEAT1*

**Figure 5:**
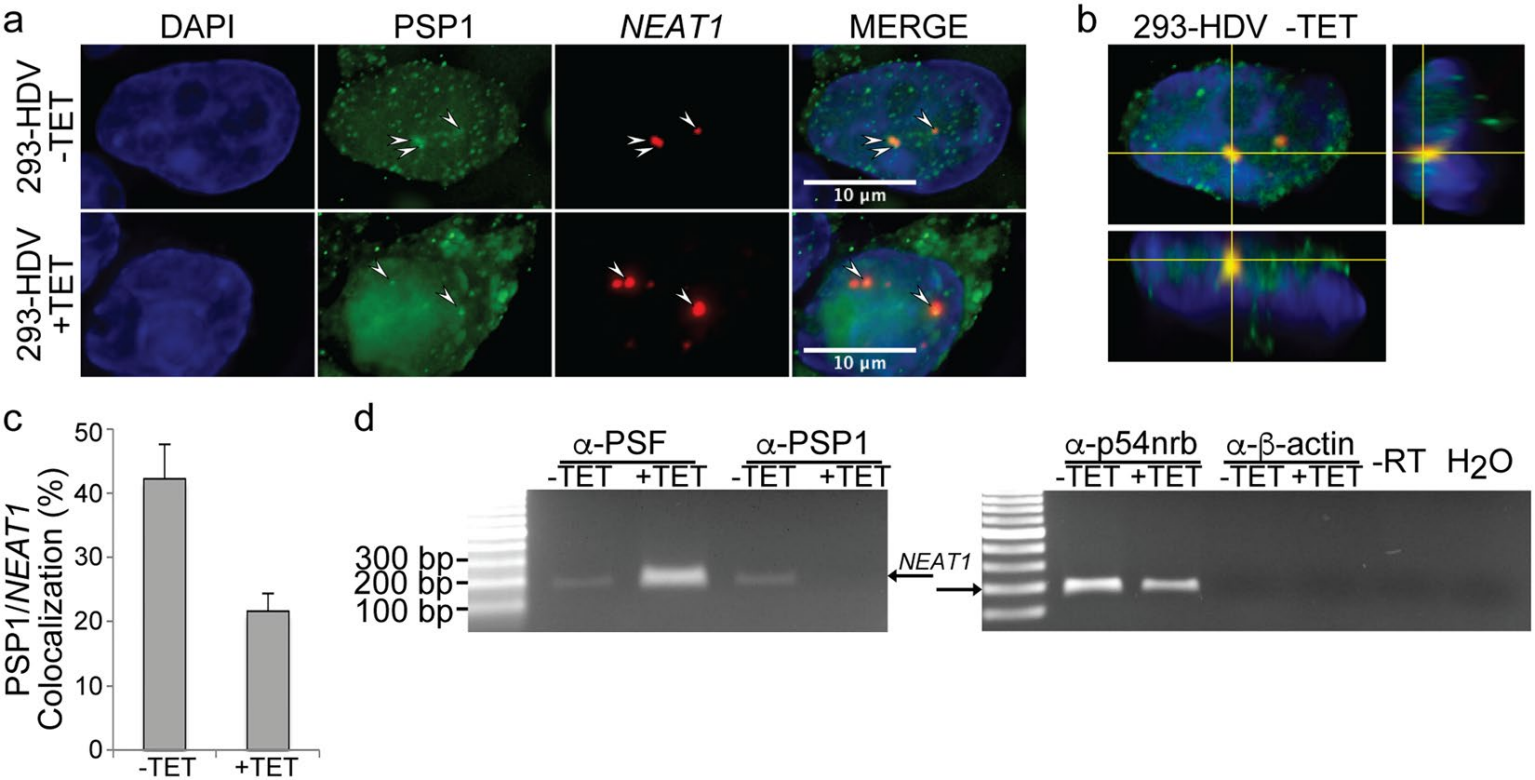
HDV replication decreases PSP1 co-localization with *NEAT1*. (**a**) Co-localization of PSP1 and *NEAT1* foci in 293-HDV cells treated or not with tetracycline, as demonstrated by immunostaining using an antibody against PSP1 (green) and *in situ* hybridization for *NEAT1* (red). DAPI (blue) was used to visualize the nucleus. Arrows indicate foci where PSP1 co-localized with *NEAT1*. (**b**) Orthogonal view of the 293-HDV cell not treated with tetracycline cell displayed in (**a**), as demonstrated by immunostaining using an antibody against PSP1 (green) and *in situ* hybridization for *NEAT1* (red). DAPI (blue) was used to visualize the nucleus. (**c**) Percentage of *NEAT1* foci co-localizing with PSP1 foci in 293-HDV cells treated or not with tetracycline. Average and standard deviation was calculated on 25 randomly selected cells per condition from two independent experiments. (**d**) Decrease of the interaction between *NEAT1_2* and PSP1 upon HDV replication in HEK-293 cells. 293-HDV cells were treated (+) or not (−) with tetracycline and the complexes cross-linked with formaldehyde. The lysates were used for RIP using α-PSF and α-PSP1 antibodies, and the α-β actin antibody was used as a negative control. The isolated RNA was reverse-transcribed with random primers and amplified by PCR with primers targeting the NEAT1_2 gene. The resulting PCR product was resolved on an agarose gel. The 100 bp DNA Ladder (NEB) was used as marker.

It is proposed that the long non-coding RNA *NEAT1* serves as a scaffold for the formation of paraspeckles^36^. Since it is required for the formation of paraspeckles, we performed an RNA-FISH on *NEAT1* along with an immuno-staining of PSP1 to investigate whether *NEAT1* remained in the nucleus. Additionally, we investigated if the relative co-localization of *NEAT1* and PSP1 changes in cells replicating HDV. Under our condition, *NEAT1* foci remain in the nucleus during HDV replication (Fig. 5a). Without induction of HDV replication, about 40% of the PSP1 foci co-localized with *NEAT1*, consistent with previous studies (Fig. 5a–c)^28–30^. When the cells were treated with tetracycline to induce HDV replication, we detected a two-fold decrease in PSP1/*NEAT1* co-localization (Fig. 5a and c). This decrease also supports our observation that PSP1 delocalizes from the nucleus to cytoplasmic PABP-containing granules. Additionally, because PSP1 is still present in the nucleus and co-localizes with *NEAT1* at a lower frequency, this result is consistent with our observation of their association upon induction of HDV replication.

To confirm the decrease of PSP1 interaction with *NEAT1* upon HDV replication, we performed RIP assays, as described above. 24 hours after induction of HDV replication with tetracycline, the cells were treated with formaldehyde to cross-link the RNA-protein complexes. Following disruption of the cells, the ribonucleoprotein complexes were immunoprecipitated with an α-PSP1 antibody, α-PSF and α-p54nrb antibodies as positive controls, or an α-β-actin antibody as an unrelated antibody. The cross-links were reversed by heating the samples, and the resulting RNA used for RT-PCR with primers designed to amplify a fragment of 200 base pairs derived from *NEAT1_2*. For the cells where HDV replication was not induced, the immunoprecipitation assay performed using α-PSP1, α-PSF or α-p54nrb antibodies showed bands corresponding to the expected size for the *NEAT1_2* amplicon (Fig. 5d). This result is in agreement with the reported interaction of *NEAT1_2* with these three proteins in paraspeckles. We did not observe this band with the negative control antibody (i.e. using the α-β-actin antibody), indicating that the band observed is not due to a non-specific binding of *NEAT1_2* with the protein G agarose beads or the antibody used for the RIP (Fig. 5d). For the cells where HDV replication was induced, no band corresponding to the *NEAT1_2* amplicon was observed when the RIP was performed with the α-PSP1 antibody (Fig. 5d). Estimated values for the relative binding of *NEAT1_2* by PSP1 using non-normalized differences of the Ct values after qPCR indicated that *NEAT1_2* interaction with PSP1 is decreased by about 5-fold, which is in agreement with our results showing that PSP1 delocalizes from the nuclear paraspeckles upon HDV replication. In contrast, bright bands corresponding the *NEAT1_2* amplicon was detected when either α-PSF or α-p54nrb antibodies were used (Fig. 5d). Estimated values for the relative binding of *NEAT1_2* by PSF suggest that there is a 7-fold increase of amount of *NEAT1_2* interacting with PSF. This could be due to an increased ability of *NEAT1_2* to interact with PSF or to higher levels of *NEAT1_2* transcripts.

### Change in expression levels of *NEAT1* upon HDV replication in HEK-293 cells

**Figure 6:**
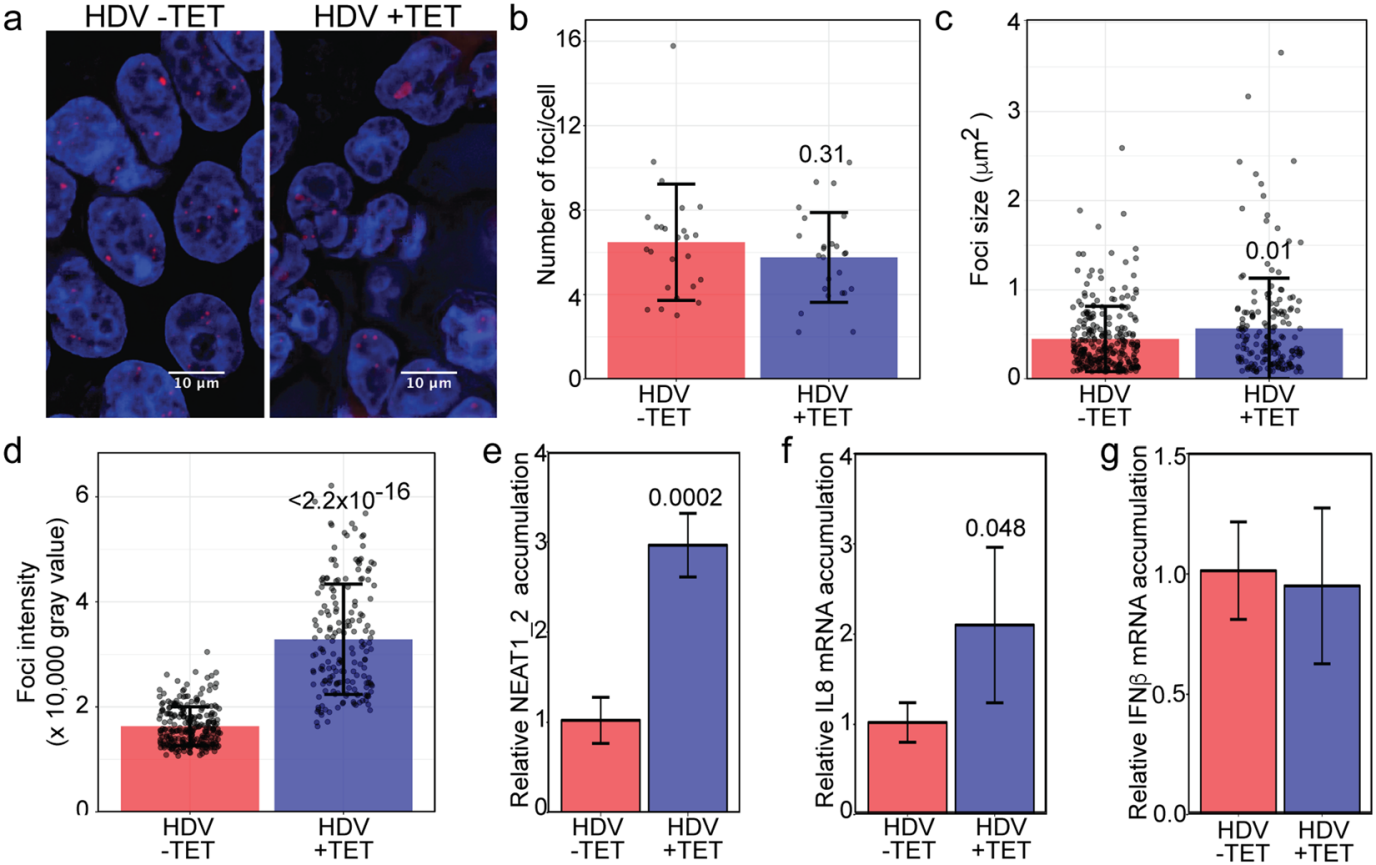
The size and intensity of the *NEAT1* foci is increased in cells replicating HDV. (**a**) NEAT1 RNA was detected by FISH in 293-HDV cells treated or not with tetracycline and the nucleus was stained by DAPI. Number (**b**), size (**c**) and intensity (**d**) of the NEAT1 foci in the two populations. (**e**–**g**) Quantification by RT-qPCR of the *NEAT1_2*, IL8 and IFNβ mRNA levels normalized to the amount of β-2-microglobulin mRNA. Values represent the mean and standard deviation of three biological replicates. Unpaired two-tailed t-tests were performed (p-values are indicated above the bars).

To assess whether *NEAT1* foci are affected by HDV replication, RNA-FISH on *NEAT1* was performed and the foci quantified (Fig. 6a). Our analysis indicates that the number of *NEAT1* foci does not vary significantly upon induction of HDV replication (Fig. 6b). However, we observed that, in cells replicating HDV, the *NEAT1* foci were larger (Fig. 6c) and their intensity were ~2-fold increased compared to the cells without HDV replication (Fig. 6d). To independently confirm these results, we performed RT-qPCR on *NEAT1_2* RNA in 293-HDV, with or without induction. Upon induction of HDV replication, we observed an increase of approximately 3-fold of *NEAT1_2* level (Fig. 6e), which is consistent with the quantifications obtained by RNA-FISH. It was previously demonstrated that an increase of *NEAT1_2* level is often accompanied by an increase in the expression of several antiviral proteins, such as IL8^42^. To assess if a similar phenomenon could be observed in cells replicating HDV, RT-qPCR was performed on both IL8 and IFNβ mRNA, under the same condition as above. Our results indicate that IL8 mRNA level is increased by approximately 2-folds and that IFNβ mRNA level is not significantly affected by HDV when compared to the uninduced cells (Fig. 6f and g). These results are consistent with previous results showing that the expression of IL8, but not IFN-β, is regulated by *NEAT1*^42^. Taken together, these results indicate that HDV replication causes an up-regulation of *NEAT1_2* and enlargement of *NEAT1* foci in this cellular system.

## Discussion

Several cellular proteins associating with HDV RNA have been previously identified^23,24,50,51^. However, only a few interactions have been confirmed in a cellular system replicating the HDV RNA genome, and a role for most of those proteins in HDV biology is still unknown. To identify proteins involved in HDV replication, we previously identified PSF and p54nrb as host proteins interacting with segments of the HDV RNA genome possessing promoter activity, both *in vitro* and in cells replicating HDV^23,24^. Because both of these proteins are part of paraspeckles, we hypothesized that components of these nuclear foci might have a role in HDV replication, and consequently that paraspeckles might be disrupted by HDV. In the current investigation, we established that three paraspeckles proteins, PSF, p54nrb and PSP1, associate with the HDV RNA genome and are required for HDV replication in HEK-293 cells. Furthermore, we determined that HDV replication induces the delocalization of PSP1 to cytoplasmic foci containing PABP, increases *NEAT1_2* level and enlarges *NEAT1* foci.

Knockdown of PSF, p54nrb or PSP1 inhibits HDV replication. PSF and p54nrb are involved in multiple RNA processes^26^, while the cellular role of PSP1 remains largely unknown and its involvement in RNA pathways remains to be characterized. These proteins also affect the expression of other proteins involved in multiple processes such as cell cycle arrest, regulation of RNA transport and stabilization, changes in transcripts and protein expression, and regulation of the cellular immunity cytokines^27^. For instance, PSF acts as transcription repressor for several genes, and its titration results in an overexpression of these genes^52^. Therefore, we cannot exclude the possibility that the reported decrease of HDV levels upon knockdown of PSF, p54nrb or PSP1 could be indirect and caused by the deregulation of other genes, RNA and proteins affected by PSF, p54nrb and PSP1. However, the need for these three paraspeckle proteins in HDV replication is consistent with previous the observation that HDAg and HDV RNA foci localize to sites usually associated with paraspeckles, in the periphery of nuclear SC35-containing speckles^53,54^.

Overall our results are consistent with a cellular stress caused by HDV replication. PSP1 is usually present in the nucleoplasm and concentrated in paraspeckles^28^. Upon HDV replication in HEK-293 cells, we observed that PSP1 delocalizes outside of the nucleus as large foci co-localizing with PABP. The cytoplasmic PABP foci we observed are similar to previously described stress granules, which are cytoplasmic structures composed of RNAs and proteins forming upon stress stimuli^49^. PABP usually associates with the polyA tail of mRNA in the cytoplasm and, upon stress, a subset of PABP cytoplasmic pool is recruited to the stress granules^55,56^. In the cellular system used, induction of HDV replication leads to cellular morphological changes, to the blockage of the majority of cells in the G1/G0 phase, and to a loss of cell adherence after two days^47^. This effect is caused by HDV replication, as it is not observed in 293 or 293-Ag cells upon addition of tetracycline^47^, and is consistent with a cellular stress caused by HDV replication which might be responsible for the cell cycle arrest^47,57^. Interestingly, other studies have shown that under transcriptional arrest and under stress conditions, PSP1 re-localizes instead to the perinucleolar region^28,58^. The reason for this difference in PSP1 re-localization is unknown, but since PSP1 interacts with HDV RNA, it is possible that shuttling of PSP1 might be affected by the viral RNA or viral ribonucleoprotein complexes. We also observed that the localization of both PSF and p54nrb is not considerably affected by HDV replication. These results are consistent with previous observations that changes in the PSP1 trafficking do not affect PSF foci localization and that knockout of PSP1 does not lead to paraspeckles disruption, indicating that PSP1 is not required for the formation of paraspeckles^30,59^.

HDV replication leads to an enlargement of *NEAT1* foci and to an up-regulation of *NEAT1* transcripts. This observation is consistent with previous reports suggesting that levels of *NEAT1* RNA and/or formation of paraspeckles are part of a cellular response to stress events, such as those occurring during viral infections^37,39,41,60–62^. Interestingly, both Influenza A virus and HSV-1 infections also upregulate *NEAT1* expression causing larger paraspeckles, which correlates with the induction of a set of antiviral genes^42^. The authors proposed that increased sequestering of the transcriptional repressor PSF from host genes by *NEAT1* activates the antiviral gene IL8, and thus innate immune response^42^. In agreement with this model, our results indicate that upon induction of HDV replication, IL8 mRNA levels is increased by approximately 2-folds when compared to the untreated cells, suggesting that a similar mechanism in which induction of *NEAT1* by HDV might causes PSF sequestering and the activation of the innate immune response. High levels of *NEAT1* were also reported to be associated with the expression of several oncogenes and shown to correlate with the occurrence of hepatocellular carcinoma^63–67^. Since PSF is a transcriptional repressor, it is tempting to speculate that higher levels of *NEAT1* in HDV infected cells also perturbs the interaction of PSF with cellular promoters associated with the regulation of oncogenes. Further experiments are clearly needed to shed light on the impact of PSF sequestering by *NEAT1* on host genes regulation and on the occurrence of fulminous hepatocellular carcinomas following HDV infection^68^.

In summary, we provide evidence of a role for three paraspeckle proteins in HDV replication, (PSF, p54nrb and PSP1), and demonstrate that HDV replication delocalizes PSP1 to cytoplasmic foci containing PABP and induces an increase of *NEAT1* expression in HEK-293 cells. Although the cellular system used represent a valuable tool to study HDV replication and pathogenesis, further examination of a role for the paraspeckles components in HDV replication is needed. Using hepatocytes or infected samples might not only confirm our observation, but also provide important insights into how these foci are involved in the life cycle of HDV, its host response and the occurrence of hepatocellular carcinomas.

## Material and Methods

### Cellular system used

293-Ag and 293-HDV cells were kindly provided by John Taylor^47^. 293, 293-Ag and 293-HDV were grown at 37 °C with 5% CO2 in Dulbecco’s Modified Eagle’S Medium (DMEM) with 10% calf serum added with 200 μg/mL hygromycine and 5 μg/mL blasticidin. Viral replication was induced with addition of 1μg/ml of tetracycline.

### Quantitative RT-PCR analysis

Total RNA was extracted using TRIzol (Invitrogen) following the manufacturer recommendations, quantified by spectrophotometry at 260 nm, and rRNA integrity was assessed by native agarose gel electrophoresis. The Reverse Transcription (RT) was carried out with the iScript cDNA synthesis kit (Biorad), following the manufacturer recommendations. Random primers were used to synthesize the total cDNA. PCR was performed with the high-fidelity Deep-Vent polymerase (NEB) according to the manufacturer’s recommendations. qPCR was performed on a Chromo4 Real-Time Detector (Bio-Rad) using the iQ SYBR Green Supermix (Bio-Rad) and the following primers: HDV-RBZ-3 Forward, 5′-CCCTCGGTAATGGCGAATG-3′; HDV-RBZ-3 Reverse, 5′-CCCAGTGAATAAAGCGGGTT-3′; INFβ Forward, 5′-CGCCGCATTGACCATCTA-3′; INFβ Reverse, 5′-GACATTAGCCAGGAGGTTCTCA-3′; β-2-microglobulin Forward, 5′-GGCTATCCAGCGTACTCCAA-3′; β-2-microglobulin Reverse, 5′-TCACACGGCAGGCATACTC-3′; NEAT1_2 Forward, 5′-GATCTTTTCCACCCCAAGAGT-3′^42^; NEAT1_2 Reverse 5′-CTCACACAAACACAGATTCCA-3′^42^. The 2^−ΔΔCt^ method was used to estimate relative cDNA levels and gene expression was normalized to β-2-microglobulin and compared to the gene expression in cells non-replicating HDV^69^. Primers efficiency curves were generated in order to ensure that the primers had similar amplification efficiencies. Each experiment was performed in technical triplicates and in three independent biological assays. The mean, standard deviation and unpaired two-tailed Student’s t-tests were calculated in R.

### Protein knockdown

Knockdown of PSF, p54nrb and PSP1 was performed using commercially available pools of specific siRNAs directed against PSF mRNA (#sc-37007; Santa Cruz), p54nrb mRNA (#sc-38163; Santa Cruz) and PSP1 mRNA (#sc-76279; Santa Cruz). As negative controls, transfection with a “scrambled” siRNA (#sc-37007; Santa Cruz), no treatment and a mock control (H_2_O) were used. Transfection was performed with Lipofectamine 2000 (Invitrogen) according to the manufacturer’s recommendations with a ratio of 80 picomoles siRNA: 5 μL lipofectamine: 100 μL DMEM. Twenty-four hours post transfection, HDAg-S expression was induced by addition of 1 μg/mL tetracycline. Twenty-four hours post-induction, the total cellular lysates were centrifuged and separated in two samples. For one sample, the total RNA was extracted using TRIzol (Invitrogen). The RNA was used for RT-PCR and RT-qPCR with primers to amplify *NEAT1*and HDV. For the other samples, the total proteins were suspended in Laemmli 4x buffer (4% SDS, 10% 2-mercaptoethanol, 20% glycerol, 0.004% bromophenol blue, 0.125 M Tris-HCl pH 6.8), and the knockdown of the proteins was confirmed by Western Blotting.

### Ribonucleoprotein immunoprecipitation assay (RIPA)

The RIP analyses were performed as reported previously^23^. Briefly, 293-HDV cells were seeded and after twenty-four hours, HDV replication was induced by addition of 1 μg/mL tetracycline. Twenty-four hours later, 1% of formaldehyde was added to cross-link the ribonucleoprotein complexes and the reaction was quenched with 0.25 M glycine, pH 7. The cells were centrifuged at 3000 rpm for 4 min and washed twice with cold PBS, and then suspended in RIPA buffer (50 mM Tris–HCl, pH 7.5, 1% Nonidet P-40 (NP-40), 0.5% sodium deoxycholate, 0.05% SDS, 1 mM EDTA, 150 mM NaCl) supplemented with 1% protease inhibitor cocktail for mammalian extracts (Sigma-Aldrich) and 1 μg RNAse inhibitor (Biobasic). The cells were mechanically lysed by pipetting on ice, followed by agitation for 30 min at 4 °C. The cellular lysate was cleared by centrifugation at 20 000 rpm for 20 min at 4 °C. Then, the supernatant was sonicated and the lysates stored at −80 °C. Co-immunoprecipitation was performed with the Protein G immunoprecipitation kit (Sigma-Aldrich). 5 μg of antibody was added to the immunoprecipitation spin columns containing 50 μL of prewashed dynabeads. The antibodies used were targeted against PSF (#B92, Abcam), PSP1 (#SAB4200067, Sigma-Aldrich), p54nrb (#05–950 Upstate) and Beta Actin (mouse monoclonal, #6276, Abcam). The antibody-dynabeads complex was incubated on a rotator overnight at 4 °C and then washed in PBST buffer (PBS with 0.02% Tween). 500 μL of cellular lysate was added to the dynabeads-antibody complex and incubated for 1 h at 4 °C, followed by 4 washes in RIPA buffer. The dynabeads-antibody-antigen complex was eluted in 200 μL of a protein storage buffer (50 mM Tris-Cl, 0.5 mM EDTA, 10 mM DTT, 1% SDS, pH7.5). In order to reverse the crosslinking reaction, the samples were heated at 70 °C for 45 min. The RNA was extracted with TRIzol (Invitrogen), ethanol-precipitated and suspended in H_2_O. The HDV and *NEAT1* RNA levels were assessed by RT-PCR.

### Sub-cellular protein fractionation

Sub-cellular protein fractionation was performed according to Suzuki *et al*.^70^, with some modifications. Briefly, the cells were wash with PSB, suspended in 1 mL of Buffer A (10 mM HEPES, 10 mM KCL, 1.5 mM MgCl_2_). 200 μl of the cell lysate was taken as “whole cell lysate” and mixed with Laemmli 4 × buffer. The rest of the lysate was incubated 10 minutes on ice, centrifuged 1 minute at 2000 × g and the supernatant was removed. The pellet was suspended in Buffer A containing 0.5 mM DTT, protease inhibitor cocktail (Roche, # RC4693116001), and 0.2% NP-40 (Igepal), incubated 2 minutes on ice and centrifuged 1 min at 2000 × g at 4 °C. The supernatant containing the “cytosolic fraction” was mixed with Laemmli 4× buffer. The pellet was suspended in NP-40 buffer (50 mM of Tris-HCL pH8.0, 0.4 M of NaCl, 5 mM EDTA pH 8.0, 1% NP-40, 0.2% SDS), incubated on ice with mixing for one hour. The lysate was then centrifuged at 4 °C, the supernatant labeled as “nuclear fraction” and mixed with Laemmli 4x sbuffer. Samples were heated 5 min at 95 °C and the samples were loaded on an sodium dodecyl sulfate polyacrylamide gel electrophoresis (SDS-PAGE) with the blueye prestain protein ladder (GeneDireX, #PM007–0500).

### Western-Blot

Bradford protein assays was performed following the manufacturer recommendations (Biorad). The protein sample from cell lysate were mixed with Laemmli 4X buffer, heated at 95 °C and migrated on polyacrylamide gel electrophoresis SDS Page (10%) in running buffer (25 mM Tris-HCl, 200 mM Glycine, 0.1% SDS). The proteins were subject to an overnight transfer to a PVDF membrane at 4 °C in 48 mM Tris, 39 mM glycine, 20% methanol, 0.037% SDS. The PVDF membrane was blocked for 1 hour at room temperature in 5% Bovine Serum Albumin (BSA) in Tris-buffered saline Tween (TBST; 200 mM Tris, 5 M NaCl, pH 7.5, 0.1% (v/v) Tween) and washed three times with TBST. The membrane was incubated with the following antibody: α-PSF (mouse monoclonal, #B92, Abcam), α-p54nrb (rabbit monoclonal, #05–950 Upstate), α-PSP1 (rabbit polyclonal, #SAB4200067, Sigma-Aldrich), α-Beta-actin (mouse monoclonal, #6276, Abcam). After three washes with TBST, the membranes were incubated with the appropriate secondary antibody in TBST with 3% BSA. The secondary antibodies used were the rabbit anti-mouse IgG HRP (polyclonal, # ab6728, Abcam), goat anti-rabbit IgG HRP (polyclonal, #ab6721, Abcam). The blots were visualized using ECL reagent according to the manufacturer’s recommendations (Thermo Scientific #32106). The membrane was then exposed to a photosensitive film and the film was scanned. The densitometric measurement was performed for some digitized blots using Image-J software. Each experiment was performed in three independent assays.

### Fluorescence Immunostaining

Cells were plated in cell culture dishes with glass bottom (Ibidi). Immunostaining was performed forty-eight hours after the induction of HDV replication with 1 μg/mL tetracycline. The cells were fixed with 4% paraformalhehyde in 1x PBS, washed three times in PBS containing 1% BSA and permeabilized with 0.5% Triton X-100. The cells were incubated with the following primary antibodies diluted in PBS containing 1% BSA: mouse monoclonal PSF (#B92, Abcam), rabbit monoclonal p54 (#05–950, Upstate), rabbit polyclonal antibody directed against the C-terminal domain PSP1 (#SAB4200067, Sigma-Aldrich), mouse monoclonal PABP (#SC32318, Cell Signaling). After three washes in PBS containing 1% BSA, the cells were washed with 0.1% Triton X-100 in PBS and incubated with the appropriate secondary antibodies in PBS containing 1% BSA. The secondary antibodies used were as following: polyclonal goat anti-mouse coupled to Alexa 488 (#A11001, Life Technologies), polyclonal anti rabbit coupled to Alexa 488 (# A11008, Life Technologies), polyclonal goat anti-mouse coupled to Alexa 594 (#A11005, Life Technologies) and polyclonal goat anti-rabbit coupled to Alexa 594 (#A11012, Life Technologies). After washing with 0.1% Triton X-100 in PBS, the cells were mounted in Vectashield containing DAPI (4′, 6-Diamidino-2-Phenylindole) staining. The cells were visualized using AxioImager inverted Z.1 microscope (Carl Zeiss Canada) with the objectives PLAN APOCHROMAT 40 × /0.95 and PLAN APOCHROMAT 63 × /1.4 OIL, the images were deconvolved using AxioVision Rel 4.8 and analyzed with either Axiocam or ImageJ. The immunofluorescence pictures shown are the combination of at least 25 Z stacks. Each experiment was performed in three independent assays.

### *In situ* hybridization

RNA fluorescence *in situ* hybridization was performed using a probe against *NEAT1* RNA (#SMF-2036-1Stellaris, Biosearch Technologies), using the protocol of the manufacturer for the simultaneous FISH and Immunofluorescence protocol was followed with minor modifications. Cells were plated in cell culture dishes with glass bottom (Ibidi). Forty-eight hours after the induction of HDV replication with 1 μg/mL tetracycline, the cells were washed twice in PBS and fixed with 4% paraformalhehyde in 1x PBS, washed three times in PBS containing 1% BSA and permeabilized with 0.1% Triton X-100. After washes in PBS, the cells were incubated with a polyclonal primary rabbit antibody directed against the C-terminal domain of PSP1 in PBS containing 1% BSA. After three washes in PBS with 0.1% Triton X-100 and the cells were incubated with the secondary antibody anti-rabbit coupled to Alexa 488 (Life Technologies). The cells were washed with PBS and incubated in the fixation buffer (3.7% formaldehyde in 1X PBS). After two washes in PBS, the cells were incubated in the wash buffer (10% formamide in 2x SSC) for 5 min at RT. Then, the cells were incubated overnight, at 37 °C, in the dark, in hybridization buffer (100 mg/mL dextran sulfate, 10% formamide in 2x SSC) containing 25 μM of NEAT1 probe. Then, the cells were washed in the hybridization buffer and they were mounted in Vectashield containing DAPI. The cells were visualized using AxioImager inverted Z.1 microscope (Carl Zeiss) with the objectives PLAN APOCHROMAT 40X /0.95 and PLAN APOCHROMAT 63X /1.4 OIL, the images were deconvolved using AxioVision Rel 4.8 and analyzed with either Axiocam or ImageJ. The immunofluorescence pictures shown are the combination of at least 25 Z stacks. Each experiment was performed in three independent assays.
